# The psychosocial impact of surgical complications on the operating surgeon: A scoping review

**DOI:** 10.1016/j.amsu.2021.102530

**Published:** 2021-07-03

**Authors:** Manjunath Siddaiah-Subramanya, Henry To, Catherine Haigh

**Affiliations:** a Department of Upper GI Surgery, Queen Elizabeth Hospital, Birmingham, UK; b University of Melbourne, Melbourne, Australia; c Department of General Surgery, Northern Hospital Epping, Melbourne, Australia; d Monash Rural Health Gippsland, Monash University, Traralgon, Australia

**Keywords:** Surgeon, Complication, Psycho, Emotion, Reaction, Surgeon wellness (term ‘psycho’ allowed us to obtain broader results including psychological and psychosocial)

## Abstract

**Background and Aim:**

Surgical complications are common, and their management is an integral part of surgical care. The impact on the surgeon, the “second victim” is significant, particularly in terms of psychological health. The aim of this review is to describe the nature of psychosocial consequences of surgical complications on the surgeons involved.

**Method:**

Following scoping review protocols, we set out to identify the evidence-base for psychosocial consequences on the operating surgeon, predominantly general surgeons, following surgical complications.

**Results:**

This scoping review identified 19 articles, mainly survey and interview based (n = 8), with all but one article from first world countries. Seven articles reported on negative emotions or depressive behavioural responses. All original studies reported on difficulty in coping (37.5%), and a range of behaviours. There was little evidence for support structures or active interventions to aid the surgeon post complication.

**Conclusions:**

The review suggests that the psychosocial impact, following a complication, is variable but affects every surgeon irrespective of the level of impact on the patient. The main variables differentiating impact are severity, and outcome of the complication and seniority of the surgeon. Reported emotions and behaviours were generally negative and persist across the surgeon's journey towards recovery. Surgeons who manage stress well exhibit largely constructive behaviours and actively work to recover. Identification of variables underpinning complications, and affected surgeons is paramount, as is the provision of services to support recovery. Efforts should be made to proactively prevent complications, via education, awareness and to formalise support processes.

## Highlights

• Surgical complications negatively impact the operating surgeon (the second victim).
• Negative emotions and behaviours manifested may persist throughout surgeon’s career.
• Surgeons’ reaction can negatively influence social and professional relationships.
• Range of factors affect coping and recovery, and the support sought tend not to focus on psychosocial aspects of coping.
• Need to develop management resources for surgeons to manage the psychosocial impact.

## 1 Introduction

Surgical complications are common in the hospital system and estimates of their frequency range from 8 to 12% across the world [[1], [2], [3], [4]]. Fortunately, not all incidents have a clinical impact due to robust hospital protocols and safety nets [5,6]. Nonetheless, incidents leading to complications are a constant concern for operating surgeons, and although anticipated and discussed with patients, complications and their impact on surgeons are not commonly discussed with colleagues or team members or studied [[7], [8], [9], [10]].

Surgery is interventional, and surgeons are particularly affected by any associated complications because of their direct involvement with the patient, whatever the outcome. Consequences for the surgeon, termed the “second victim” [11] in this context, have been reported to have a broad personal impact [10,12]. The effects may be physiological, physical, emotional, or behavioural. The origin and perpetuation of the cause and effect of these surgical complications has been shown to have an association with a number of factors such as long working hours, conflicts at home or with colleagues, administrative stressors, training responsibilities, and poor physical health of the surgeon [9,13]. There are few reported reviews of the extent, root cause or needs analysis of these issues.

Therefore, we aim to conduct a scoping review to understand the magnitude and nature of the psychosocial consequences of surgical complications for the operating surgeon along with coping mechanisms utilised. We contend this should be the first step in understanding the journey of a surgeon from the incident to their psychological recovery, with an ultimate aim to architect an approach to prevention, recognition, and support so that recommendations can be made to various training boards, hospital employers, colleges and policy makers.

## 2 Methods

A scoping review protocol was used, which is a form of review methodology that addresses key concepts, types of evidence, and gaps in the literature by systematically searching, selecting, and synthesizing existing knowledge [14]. Using the principles and framework proposed by Arksey et al. [15], we employed the following five phases: 1) identifying the research question; 2) identifying potentially relevant articles; 3) selecting articles; 4) charting the data; and 5) reporting the results.

The research team comprised members with backgrounds in surgery, psychology, and surgical education and training, and considered all facets of psychosocial consequences to obtain an overall impression of how a surgeon is impacted. The broad primary research question was ‘what are the psychosocial consequences of surgical complications on the operating surgeon?’ with a secondary question being ‘what are the coping mechanisms that surgeons utilise and what are their typical reactions to complications?’

The initial search was conducted by the primary investigator (MS-S) using Ovid Medline with input from co-investigators (HT) and (CH) applying the key terms mentioned *(*Appendix 1*).* The term “complication” is broadly used and poorly defined. Therefore, in our study, we have also included search terms that represent the concept of complication such as error, treatment failure and adverse event. As Wu et al. described the “second victim” phenomenon in 2000, we searched for articles after this publication [11]. Subsequent searches were undertaken using Web of Science, Embase, Scopus, PsychINFO, Educational Resources Information Centre (ERIC) and Cumulative Index to Nursing and Allied Health Literature (CINAHL).

Endnote X9 (Version 9.3.3 – Thomas Reuters, New York) was used to import all citations. Further screening used the following inclusion criteria:

a) Reported on psychosocial consequences irrespective of the timing or outcome of the complication
b) Focused on general surgeons so they formed the majority of the participants

The charting approach was an iterative process involving data extraction of specific features and themes in line with descriptive analysis [16]. General and specific characteristics relevant to our study were obtained, focussing on thematic datapoints with the end goal of developing a construct for future application.

## 3 Results

**Fig. 1:**
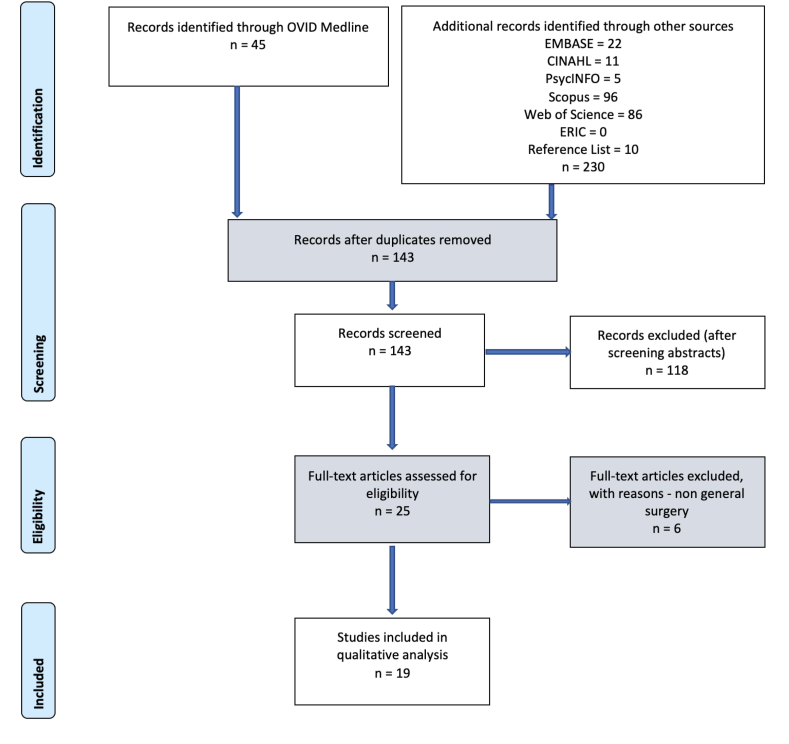
PRISMA Flow Chart for method of identification and selection of the articles reviewed.

The search resulted in 19 articles that met the selection criteria (Fig. 1). The results are presented in Table 1. A full list of the articles included is listed in *(*Appendix 2*)*

**Table 1:** General Characteristics of the Studies

Year of Publication	2010–2020 (Luu, Leung et al., 2012, Luu, Patel et al., 2012, Varjavand, Nair et al., 2012, Pinto, Faiz et al., 2013, Pinto, Faiz et al., 2014, Marmon and Heiss 2015, Turner, Johnson et al., 2016, Bunni 2017, Han, Bohnen et al., 2017, Schroeder 2018, Bohnen, Lillemoe et al., 2019, Joliat, Demartines et al., 2019, Srinivasa, Gurney et al., 2019, Biggs, Waggett et al., 2020, Pellino and Pellino 2020, Tebala 2020)	2000–2010 (Iribhogbe 2010, Patel, Ingalls et al., 2010, Shanafelt, Balch et al., 2010)	
**Country of Origin**	**High-Income** ***UK*** (Pinto, Faiz et al., 2013, Pinto, Faiz et al., 2014, Turner, Johnson et al., 2016, Bunni 2017, Biggs, Waggett et al., 2020, Tebala 2020) ***USA*** (Patel, Ingalls et al., 2010, Shanafelt, Balch et al., 2010, Varjavand, Nair et al., 2012, Marmon and Heiss 2015, Han, Bohnen et al., 2017, Bohnen, Lillemoe et al., 2019) ***Canada*** (Luu, Leung et al., 2012, Luu, Patel et al., 2012) *New Zealand* (Srinivasa, Gurney et al., 2019) ***Italy*** (Pellino and Pellino 2020) ***Germany*** (Schroeder 2018) ***Switzerland*** (Joliat, Demartines et al., 2019)	**Low-Income** ***Nigeria*** (Iribhogbe 2010)	
**Source of Article**	**OVID Medline** (Luu, Leung et al., 2012, Marmon and Heiss 2015, Turner, Johnson et al., 2016, Bunni 2017, Schroeder 2018, Bohnen, Lillemoe et al., 2019, Joliat, Demartines et al., 2019, Srinivasa, Gurney et al., 2019, Pellino and Pellino 2020, Tebala 2020)	**Scopus** (Iribhogbe 2010, Shanafelt, Balch et al., 2010)	**Reference List** (Patel, Ingalls et al., 2010, Luu, Patel et al., 2012, Varjavand, Nair et al., 2012, Pinto, Faiz et al., 2013, Pinto, Faiz et al., 2014, Han, Bohnen et al., 2017, Biggs, Waggett et al., 2020)
**Journal Source**	**Surgical** (Patel, Ingalls et al., 2010, Shanafelt, Balch et al., 2010, Luu, Leung et al., 2012, Pinto, Faiz et al., 2013, Pinto, Faiz et al., 2014, Marmon and Heiss 2015, Turner, Johnson et al., 2016, Bunni 2017, Han, Bohnen et al., 2017, Schroeder 2018, Bohnen, Lillemoe et al., 2019, Joliat, Demartines et al., 2019, Srinivasa, Gurney et al., 2019, Biggs, Waggett et al., 2020, Pellino and Pellino 2020)	**Medical** (Iribhogbe 2010, Tebala 2020)	**Educational** (Luu, Patel et al., 2012, Varjavand, Nair et al., 2012)
**Type of Study**	**Original** (Iribhogbe 2010, Patel, Ingalls et al., 2010, Shanafelt, Balch et al., 2010, Luu, Patel et al., 2012, Pinto, Faiz et al., 2013, Pinto, Faiz et al., 2014, Han, Bohnen et al., 2017, Biggs, Waggett et al., 2020)	**Perspective** (Luu, Leung et al., 2012, Varjavand, Nair et al., 2012, Marmon and Heiss 2015, Turner, Johnson et al., 2016, Schroeder 2018, Bohnen, Lillemoe et al., 2019, Tebala 2020) Editorial (Bunni 2017) Letter (Pellino and Pellino 2020)	**Systematic Review** (Joliat, Demartines et al., 2019, Srinivasa, Gurney et al., 2019)
**Study Design**	**Semi-structured Interview** (Luu, Patel et al., 2012, Pinto, Faiz et al., 2013)	**Web or Paper-based Survey** (Iribhogbe 2010, Patel, Ingalls et al., 2010, Shanafelt, Balch et al., 2010, Pinto, Faiz et al., 2014, Han, Bohnen et al., 2017, Biggs, Waggett et al., 2020)	

The general features (Table 1) were that most of the articles were published between 2011 and 2020 (n = 16, 84%) and the majority of the studies came from the United Kingdom (UK) (n = 6, 31.5%) or the United States of America (USA) (n = 6, 31.5%). All but one of the studies were from western countries. The majority of the articles were from surgical journals (n = 15, 79%) with input from medical (n = 2, 10.5%) and educational journals (n = 2, 10.5%). There was a range of article types depicting the heterogeneity of the literature available. Original studies (n = 8, 42%) took an exploratory approach targeting individual surgeons, either via semi-structured interviews [17,18] or anonymous surveys [1,5,[19], [20], [21], [22]]. Perspectives were the predominant opinion-based article type [6,[23], [24], [25], [26], [27], [28]]. There were two systematic reviews [29,30], that differed from our scoping review which aimed to provide an up-to-date evidence base, identifying gaps and providing directions for interventions.

The majority of the articles included surgeons from multiple specialities. All original studies explored complications of varying severities, but only three articles discussed a “serious” complication that was reported but poorly defined [18,19,22]. No studies used the Clavien-Dindo severity classification for complications [31].

Importantly, the timespan between the complication and research varied which may have impacted any potential recall bias for the incident. Two studies collected data in the immediate aftermath (within 3 months) of the complication [17], showing over 30% of surgeons had experienced a complication within this time frame [1]. Two of the studies were conducted in the early phases following a complication with the majority of participating surgeons (>80%) reporting a complication within 12 months prior to the study [1,19].

Table 2 shows some of the specific features related to the emotions and behaviours reported in the selected studies. Negative emotions or behavioural responses were reported by all studies. Three of the original studies discussed negative impacts extending to the surgeon's social life (n = 3, 37.5%) [5,17,18], while another two reported that complications negatively affected the surgeon's interactions with their colleagues [5,17]. Three studies reported on the behavioural impact, in that a more cautious approach to similar surgery is often adopted subsequently (n = 3, 37.5%) [17,18]. Three studies suggested that senior surgeons may be better able to cope with the stress of complications (n = 3, 37.5%) [[18], [19], [20]], the reasoning being that they either reported lower complication rates as they accumulated experience [21] or successfully concealed their emotions [19]. The specific features and their interpretations have been further elaborated in the discussion.

**Table 2:** Emotions and Behaviours Reported in the Original Studies

Authors and	Biggs et al.	Han et al.	Pinto et al.	Pinto et al.	Luu et al.	Patel et al.	Shanafelt et al.	Iribhogbe et al.
Year of Publication	2020	2017	2014	2013	2012	2010	2010	2010
Emotions								
**Depressive or Negative**			NR					NR
Guilt	Yes	Yes		Yes				
Sadness	Yes	Yes		Yes				
Crisis of confidence	Yes			Yes		Yes		
Worry for reputation	Yes			Yes	Yes			
Worry for patient	Yes			Yes	Yes			
Anxiety	Yes	Yes			Yes			
Disappointment	Yes			Yes				
Shame or Embarrassment		Yes						
Emotional exhaustion or Burnout							Yes	
Low mood							Yes	
No feeling or numbness		Yes						
Devalued or feeling of worthlessness					Yes			

								
**Aggressive**			NR		NR	NR	NR	NR
Anger	Yes	Yes		Yes				
								
**Behavioural Responses**								
**Constructive Behaviours**							NR	
Getting on with life	Yes		Yes					Yes
Taking a break								Yes
Reflective practice	Yes		Yes	Yes				
Seeking support from colleagues	Yes	Yes	Yes	Yes	Yes	Yes		
Seeking help from external support groups or psychologists		Yes	Yes		Yes	Yes		
Seeking support from family/friends	Yes	Yes	Yes		Yes	Yes		
Learning and planning following complication to improve future outcome	Yes		Yes	Yes	Yes			
Change of practice to risk aversion or with caution	Yes			Yes	Yes			
Exercise				Yes		Yes		
Actively coping			Yes					
Humour			Yes					
Seeking support form religious faith			Yes					
								
								
**Repressive or Negative Behaviours**								NR
Self-blame			Yes					
Aggressive to colleagues	Yes							
Blaming external factors	Yes			Yes				
Alcohol abuse	Yes		Yes			Yes		
Substance abuse	Yes					Yes		
Disassociation	Yes							
Self-distraction	Yes		Yes					
Internalisation	Yes							
Rumination	Yes							
Not seeking or engaging in any support		Yes		Yes	Yes	Yes		
Lack of concentration (affecting general functionality or clinical judgement)				Yes		Yes		
Not enjoying personal life				Yes		Yes		
Avoidance			Yes			Yes		
Denial			Yes					
Venting			Yes					
Aloof or withdrawn					Yes			
Protective or self-preservation					Yes			
Sensitive					Yes			
Over personalisation					Yes			
Depersonalisation							Yes	
								
								
**Physiological Responses**	NR	NR	NR				NR	NR
Feeling sick or nauseous				Yes				
Trouble with sleep					Yes	Yes		
Palpitations					Yes			
								

*NR – Not Reported.

## 4 Discussion

**Fig. 2:**
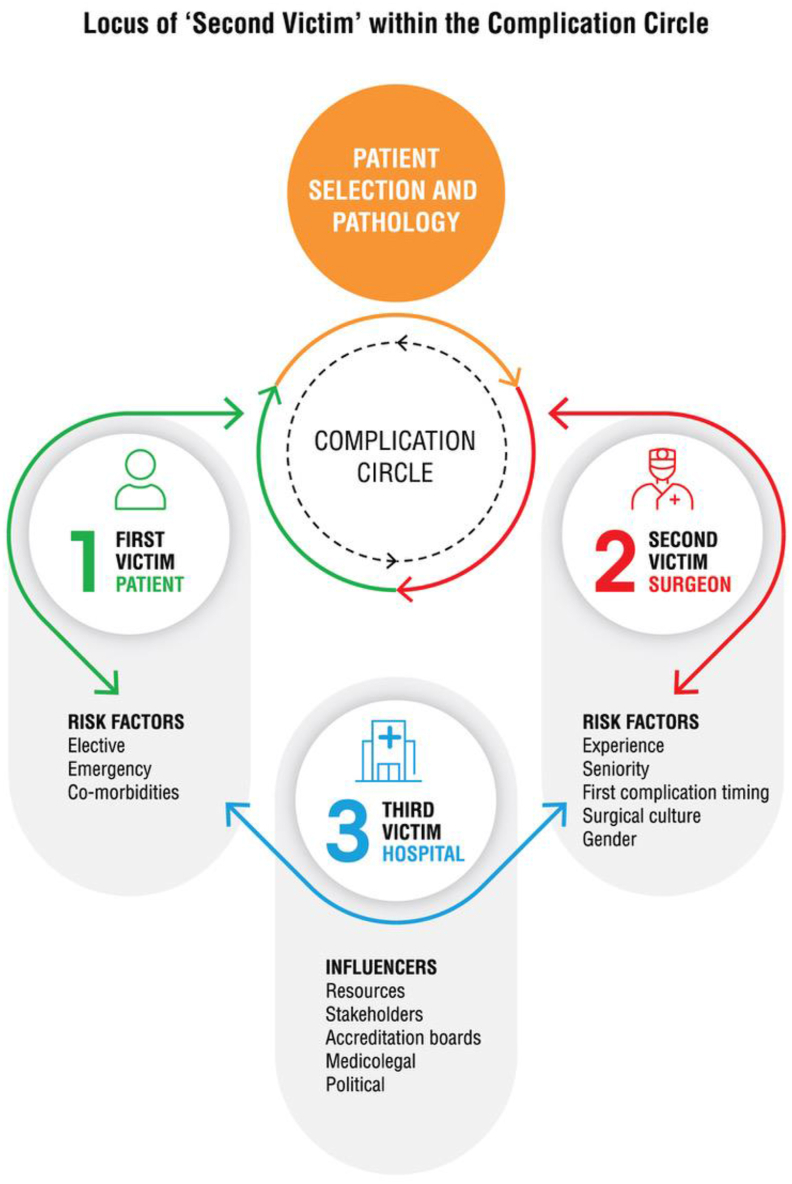
Locus of 'Second Victim' within the Complication Circle.

This is the first scoping review exploring this topic, showing that commonly occurring surgical complications induce a largely negative emotional and behavioural response for the operating surgeon that is largely unreported. Three non-technical factors are considered in the genesis of a surgical complication; the patient, the disease and the surgeon [24] *(*Fig. 2*)*, each with their own risk factors and influencers*.*

Surgeons are the second victims in the event of complications occurring for the patient (who is the ‘first victim’) intra or post-operatively, and they bear the stress of the medical management of the complication, typically receiving limited support from the treating institution (the ‘third victim’) [27] *(*Fig. 2*).*

Some surgeons appear to be at more risk of developing second victim syndrome [27]. Key factors identified include experience; attributing the complication to a lapse in judgement or concentration, lack of knowledge or skill, or errors in the healthcare system; being female, ‘burned-out’ or fatigued; feeling demoralised or unrewarded; and perceiving an imbalance between professional and personal lives [17,21,27]. Female surgeons and junior surgeons tend to personalise the situation, appear to be overtly more affected by the experience and are more open in admitting to this impact [17]. The perceived imbalance between work and personal life is reported to be overwhelming at times for these surgeons [32]. Some of these factors could be addressed by adopting and utilising a flat hierarchy within the department and good leadership plays a vital role. Understanding these factors enables us to appreciate the vulnerability of the ‘second victim’ and their psychological responses, and in turn the coping mechanism they adopt. Surgeons often feel that complications are attributed to their technical capabilities and judgement, and can be profoundly impacted irrespective of the severity of the complication [19].

The personal toll of complications is significant and often unacknowledged. Most surgeons appear to remember their first significant complication, and interviews reveal that this memory endures across their careers even as experience grows [20]. Factors which increased the psychosocial impact include the setting and outcome of the complication. Complications that occurred during elective operations, particularly when unexpected, were reported to have greater personal effect [18]. Similarly, a complication leading to death or severe disability such as loss of a limb, or paralysis resulted in a greater emotional burden on the operating surgeon [18]. This was exemplified by Patel and colleagues with 41% of the surgeons surveyed saying that the death of a patient caused significant emotional distress [20].

### 4.1 Range of emotions and reactions, and their impact

Our review provides evidence of the range of emotions that surgeons experience following a complication *(*Table 2*)* [12,33,34]. The nature of emotions included short lived “aggressive”, and “depressive” emotions which were commonly long-lasting, affecting other facets of daily life. Surgeons stereotypically strive to be perceived as strong and unemotional [35], but are actually greatly affected by even the perception of committing an error, experiencing stress and anxiety as a result [28]. Luu, Patel et al. (2012) reported that senior surgeons disclosed similar and profound emotions while managing to maintain a composed external appearance. In contrast, a survey of 7905 surgeons [21] reported no difference in reactions to perceived errors by seniority but this report did not explore future or long-lasting effects.

Depressive emotions are more often reported and include concern for the patient (91.5%), guilt (64.6%), anxiety (68.3%) and disappointment (63.4%) [5]. Similarly, in another survey-based study by Han et al., most of the surgeons reported feeling guilty (60%), anxious (66%), sad (52%), ashamed or embarrassed (42%) with relatively fewer revealing anger (29%) [19]. Intense depressive and negative emotions are more common compared to transitory aggressive reactions such as anger.

Emotions are experienced at all levels of seniority [19]. However, in one survey, 79% of surgeons with 10+ years of experience reported having no negative feelings or feeling numb post complications. The same study observed that the incidence of emotions reported was higher earlier in the surgeons’ career and then rose again approaching retirement. Shanafelt et al. similarly, reported that older surgeons were less likely to report complications which tended to decrease by approximately 15% for every decade of age, an inverse correlation with skill and experience. Whether this is just a reduced tendency to report or a true decrease in feelings of guilt and self-blame is unclear. Older surgeons may also experience cognitive dissonance between the psychosocial experience of a complication and the surgical stereotype of the powerful in-control individual and this might explain the apparent reduced impact [29]. In addition, senior surgeons may have access to a better professional support structure.

The emotional impact of complications affects help-seeking. For example, concerns for one's reputation lead to behavioural changes such as a reduced tendency to seek help, reluctance to speak up about complications and fewer constructive interactions with colleagues [36]. These may extend to and negatively impact the surgeon's family life, affecting another source of support [5].

For major complications, emotions are sometimes so strong that surgeons are at high psychological risk. Pinto el al. Studied emotional and behavioural change following poor patient outcomes and reported that 36.2% of surgeons experienced degrees of acute traumatic stress [1]. Furthermore, short-term emotional exhaustion or feelings of numbness often followed major surgical complications, with these emotions often appearing within three months, doubling the risk of surgeons developing major depression [21].

Emotions following a surgical complication, although varied, were predominantly negative potentially affecting surgeons for a prolonged period of time over their career. Emotional changes are experienced by all surgeons irrespective of gender, age and experience. The most concerning outcome of these negative emotions, at least in the initial phases following complication, was the reluctance or inability of the surgeons to seek help which may further prolong the duration of their journey towards recovery and in certain cases lead to major psychiatric effects, all of which clearly require support and intervention.

### 4.2 Behavioural responses depicting coping strategies change over time

Surgeons possess a range of traits which enable them to cope with stressors [26], and responses to complications vary [18]. Behavioural responses are either constructive, e.g., planning to improve future patient outcomes [1,5,17], or repressive impacting negatively on personal and family life (54.9%), or the workplace (25.6%) [5]. There are likely to be elements of both of these behaviours over time (Table 3)*.*

In a web-based survey, the majority of participants reported constructive behaviours post complication, but also adopted defensive practices with 63% becoming more cautious and 43% ruminating [5]. It is unclear how long these behaviours persisted after the complication. An interview-based study [36] reported similar outcomes. Surgeons’ responses are sensitive to public and medicolegal reactions to complications [37], in turn encouraging defensive practice [37,38]. Medicolegal issues [37] can have a reputational impact, and in some countries personal threats to surgeons have been reported [39]. These effects further perpetuate defensive behaviours [24].

Once the patient's outcome is being managed surgeons typically seek support from friends, family or colleagues [5,[17], [18], [19], [20]] and professional circles [1,17,19,20]. Biggs et al. noted that most surgeons (81.7%) discussed the technical aspects of cases with their colleagues and engaged with patients and families (57.3%) through open disclosure. Some surgeons choose proactive avenues such as exercising [18,20], humour [1] and hobbies [1,5,22], whereas others take leave [1,5,18] or use religion for solace [1].

Repressive or negative behaviours were reported in the immediate aftermath of complications. Harmful substance usage was reported in a minority (10% in the study by Biggs et al. and 6.5% of those surveyed by Patel et al. [5,20]. Biggs and colleagues, reported that 7% of the surgeons demonstrated a tendency towards dissociation [5], which could take various forms e.g., minimising social interactions [17], avoidance [1,20], remaining aloof and withdrawing [17], internalisation, rumination, self-distraction, and denial [1,5]. These behaviours were considered harmful if prolonged. Persistent self-distraction was reported as one of the three factors associated with acute traumatic stress [40,41]. Self-blame, was noted to a lesser extent (22% [5], presenting as identifying a lapse in judgement, a lack of knowledge and/or a loss of concentration. Lapses in judgement were noted more frequently when considering major complications (31.8%) while lack of knowledge was perceived to be the issue for 4.5% of surgeons [21]. Biophysiological symptoms are not often reported and are difficult to attribute directly to specific events.

Surgeons' behaviours following a complication changed over time. The pace and nature of these changes is dependent on a number of factors including experience, resilience and the personality of the surgeon, support from the department and external expectations [24]. Over the years, a number of models have been developed to depict the phases of the second victim's journey following a complication and these have detailed illustrations on each phase [12,17,24]. Understandably, these phases are neither linear nor sequential, but intersect with various emotional and behavioural responses that may linger indefinitely across different stages.

The first response after a complication is one of confusion, denial, intense emotions and physiological reactions. The situation is chaotic and most attention is directed towards managing the patient and seeking reassurance by scotomising the event [24]. The most beneficial intervention at this stage is emotional support. The next phase is one of realisation and exploration where the surgeon appreciates the true impact of the complication [24] and thinks beyond the initial event [17]. The surgeon can reason and investigate the complication asking ‘why’ rather than ‘what’. This has been suggested as an early juncture where surgeons may be willing to accept active support if provided in a protective environment. The next phase is one of openness and readiness, where surgeons are prepared to talk and may make some important decisions, actively seeking support and professional help [12]. This is the phase where proactive and organised support, whether offered in-house or professionally, is necessary and would be most effective. The long-term effects of surgical complications may endure across the an entire career involving continuous learning and reflection resulting in ‘surgical maturity’ [17].

Seniority of the surgeon accounted for some of the intensity of the responses. Earlier in their career, especially when newly appointed, surgeons experience greater emotional impact due to adjustments to their new level of responsibility [18], and are more likely to report long lasting negative consequences [42].

The current culture in surgery was reported to emphasise the practical and technical aspects of complications, and was not conducive to the discussion of emotional and behavioural impacts [18,43], thus encouraging repression, self-defence and depersonalisation [19]. This atmosphere prevented surgeons from seeking support even when offered [20].

**Fig. 3:**
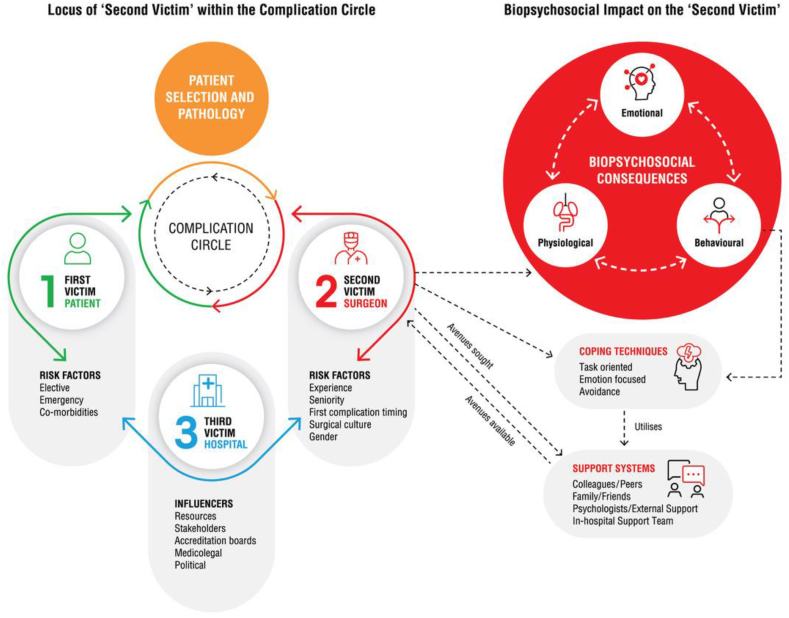
‘Second victim’ - Their influencers in the complication circle and along the pathway to recovery.

Surgeons’ responses change in their journey to achieve normalcy with constructive behaviours aimed at the patients which frequently evolve into defensive practice and repressive behaviour that is self-protective. These behaviours relate to experience, and tend to be influenced by the working environment and culture (Fig. 3).

### 4.3 Strengths and Limitations

The strengths of this study include the systematic approach [15], and broad background of the research team. We included the whole spectrum of undesired outcomes under the umbrella of complications, including all levels of severity and aimed to describe the holistic biopsychosocial impact on a surgeon following a complication.

Limitations include the bias in the literature towards high-income countries restricting generalisability. Furthermore, the focus on general surgeons makes the study less applicable to trainees and other specialities. Recall bias was a consideration as all original studies were retrospective in nature relying on surveys or interviews as the basis for information. None of the studies involved the direct observation of surgeons’ emotions or behaviour when complications occurred or in the period immediately after the complication. Nonetheless a prospective design would be challenging because of the unpredictability of the timing of complications and the undue stress that such a study may cause for the surgeon involved.

### 4.4 Gaps in literature and recommendations

• The term ‘complication’ should be operationally defined
• Consideration of prevention, education about and awareness of the psychological impact of complications in term-assessments may aid trainees to recognise symptoms early, and encourage openness to seek or receive assistance as necessary
• Proactive support has not been studied but should be offered to surgeons as they can lack insight into their responses given the immediate focus on the patient (first victim)
• When complications occur:
  o Tailored support commensurate with levels of seniority should be provided.
  o Negative behaviours should be carefully monitored by colleagues.
  o Psychosocial support should be offered to navigate medico-legal ramifications.
  o The interaction between the surgeon (second victim) and the hospital (third victim) can exacerbate negative outcomes Understanding this relationship could determine how to best benefit surgeons' well-being.
  o It is difficult to ascertain timeframes when behaviours may change. Research should address both the nature and timing of interventions to support recovery.
• Some support structures exist, but their impact and efficacy are not established. Future research could focus on developing and evaluating these at all levels, from surgical units to national licensing authorities.
• More research is required to understand the situation in low-income countries.

## 5 Conclusion

This review has found that surgical complications can have an immense impact on surgeons and can endure for a prolonged period of time. Biopsychosocial consequences for a surgeon following complications are significant and are influenced by multiple stressors. Depressive emotions are common and are longer lasting than typically perceived. Behaviours that eventuate in response to complications strongly influence whether the surgeon recovers. Surgeons who are inherently poised to manage stress well exhibit largely constructive behaviours and work towards achieving a better outcome for the patient. Recommendations include prevention, education, and active support to prepare surgeons to recognise and manage their response to complications.

## Provenance and peer review

Not commissioned, externally peer-reviewed.

## Figures and Tables

**Table:** 

Primary Author	Year of Publication	Country of Origin	Type of Study	Title	Journal
**Joliat**	2019	Switzerland	Systematic Review	Systematic review of the impact of patient death on surgeons	British Journal of Surgery
**Srinivas**	2019	New Zealand	Systematic Review	Potential Consequences of Patient Complications for Surgeon Well-being. A Systematic Review	JAMA Surgery
**Schroeder**	2018	Germany	Perspective	How Surgeons Deal with Complications	Surgical Infections
**Luu**	2012	Canada	Perspective	When Bad Things Happen to Good Surgeons: Reactions to Adverse Events	Surgical Clinics of North America
**Pellino**	2020	Italy	Letter	Deaths, errors and second victims in surgery: an underestimated problem	British Journal of Surgery
**Tebala**	2020	UK	Perspective	Is there a standard reaction of surgeons to surgical complications? Study on an interesting historical case	Medical Hypotheses
**Bohnen**	2019	USA	Perspective	When Things Go Wrong: The Surgeon as Second Victim	Annals of Surgery
**Bunni**	2017	UK	Editorial	Complications - A surgeon's perspective and humanities' methods for personally dealing with them: The “4 R's”	International Journal of Surgery
**Turner**	2016	UK	Perspective	The impact of complications and errors on surgeons	The Bulletin of the Royal College of Surgeons of England
**Marmon**	2015	USA	Perspective	Improving surgeon wellness: The second victim syndrome and quality of care	Seminars in Pediatric Surgery
**Varjavand**	2012	USA	Perspective	A call to address the curricular provision of emotional support in the event of medical errors and adverse events	Medical Education
**Biggs**	2020	UK	Original	Impact of surgical complications on the operating surgeon	Colorectal Disease
**Han**	2017	USA	Original	The Surgeon as the Second Victim? Results of the Boston Intraoperative Adverse Events Surgeons' Attitude (BISA) Study	The American Journal of Surgery
**Pinto**	2014	UK	Original	Acute traumatic stress among surgeons after major surgical complications	The American Journal of Surgery
**Pinto**	2013	UK	Original	Surgical complications and their implications for surgeons' well-being	British Journal of Surgery
**Luu**	2012	Canada	Original	Waking up the next morning: surgeons' emotional reactions to adverse events	Medical Education
**Patel**	2010	USA	Original	Collateral damage: The effect of patient complications on the surgeon's psyche	Surgery
**Shanafelt**	2010	USA	Original	Burnout and Medical Errors Among American Surgeons	Annals of Surgery
**Iribhogbe**	2010	Nigeria	Original	Attitude of Nigerian surgeons to intraoperative deaths	Nigerian Journal of Clinical Practice

## Appendix 1 Search Result from OVID Medline

1. (error* or death* or adverse event* or adverse outcome*or mortality* or complication*).ti 441106
2. (psycho* or attitude* or emotion* or burnout* or stress*or reaction* or surgeon wellness* or second victim*or well-being*).tw 2831658

## Appendix 2 List of Articles

This figure represents surgeons as the ‘second victim’ at the centre of the complication circle. It explains their position within this circle encompassing the patient (the ‘first victim’), and the health service (the ‘third victim’) and the complex interplay between these three ‘victim-players’. The focus is shown on the ‘second victim’ and this interplay would change if it were to be on ‘first’ or ‘third’ victims. All three victims are in turn potentially affected by intrinsic and extrinsic risk factors and influencers.

The figure shows all the three facets of the biopsychosocial impact of a complication on the ‘second victim’, and how these inter-relate and might influence the surgeon's adoption of a coping strategy. It demonstrates how surgeons may utilise support avenues that are available as they navigate this experience towards recovery.

## Annals of medicine and surgery

The following information is required for submission. Please note that failure to respond to these questions/statements will mean your submission will be returned. If you have nothing to declare in any of these categories then this should be stated.

## Registration of research studies

In accordance with the Declaration of Helsinki 2013, all research involving human participants has to be registered in a publicly accessible database. Please enter the name of the registry and the unique identifying number (UIN) of your study.

You can register any type of research at http://www.researchregistry.com to obtain your UIN if you have not already registered. This is mandatory for human studies only. Trials and certain observational research can also be registered elsewhere such as: ClinicalTrials.gov or ISRCTN or numerous other registries.

1.Name of the registry: researchregistry.com
2.Unique Identifying number or registration ID: reviewregistry1115
3.Hyperlink to your specific registration (must be publicly accessible and will be checked):

https://www.researchregistry.com/browse-the-registry#registryofsystematicreviewsmeta-analyses/
