# Corrigendum to “The psychosocial impact of surgical complications on the operating surgeon: A scoping review” [Ann. Med. Surg. 67 (July 2021) 102530]

**DOI:** 10.1016/j.amsu.2021.102585

**Published:** 2021-07-24

**Authors:** Manjunath Siddaiah-Subramanya, Henry To, Catherine Haigh

**Affiliations:** a Department of Upper GI Surgery, Queen Elizabeth Hospital, Birmingham, UK; b University of Melbourne, Melbourne, Australia; c Department of General Surgery, Northern Hospital Epping, Melbourne, Australia; d Monash Rural Health Gippsland, Monash University, Traralgon, Australia

The authors regret that the following sentence should have read as: There are likely to be elements of both of these behaviours over time (Table 2).

The authors would like to apologise for any inconvenience caused.

